# Deformation Control of TC4 Titanium Alloy in Thin-Walled Hyperbolic Structures During Hot Forming Processes

**DOI:** 10.3390/ma17246146

**Published:** 2024-12-16

**Authors:** Tao Zhang, Jianchao Xia, Xiuquan Han, Lihua Du, Lihua Chen, Yujie Han, Fengchao Cao, Duoduo Wang, Xiaochuan Liu

**Affiliations:** 1 School of Materials Science and Engineering, Northwestern Polytechnical University, Xi’an 710072, China; 13581842743@163.com; 2 AVIC Manufacturing Technology Institute, Beijing 100024, China; hanxq001@avic.com (X.H.); hanyujiehao@126.com (Y.H.); fengchaocao@126.com (F.C.); 3 School of Mechanical Engineering, Xi’an Jiaotong University, Xi’an 710049, China; xiajianchao@stu.xjtu.edu.cn (J.X.); liuxiaochuan2020@xjtu.edu.cn (X.L.); 4 Beijing North Vehicle Group Corporation, Beijing 100072, China; chen17601642300@163.com (L.C.); zztaurus@163.com (D.W.)

**Keywords:** TC4 titanium alloy, thin-wall hyperboloid, hot coupling forming, curved blanking drawing, deformation control

## Abstract

The thin-walled hyperbolic structures made from titanium alloy primarily encompass two typical forms: hyperbolic convex and hyperbolic concave (saddle). This paper addresses the technical challenges associated with the forming processes that frequently result in ripples or wrinkles in these configurations. Specifically, it investigates precision control techniques for the hot forming process of thin-walled hyperbolic skins from TC4 titanium alloy. The present study examines the relationship between the instability characteristics and defect features of the thin-walled hyperbolic skins, establishing a constitutive model for TC4 titanium alloy and conducting uniaxial tensile tests. For the hyperbolic convex skin and the hyperbolic saddle skin, small-margin coupled hot pressing and curved edge forming processes are employed, respectively. Results are analyzed to identify the forms and distribution patterns of forming defects across different geometries. Furthermore, the surface accuracy following the forming processes is compared, culminating in a summary of the relationship between the ratio of the sum of chord heights to the sum of chord lengths and the occurrence of ripples and wrinkles.

## 1. Introduction

In modern aerospace engineering, the significance of complex thin-walled curved surface structures [1,2] is increasingly pronounced. These structures not only fulfill essential functions such as load-bearing and thermal insulation, but they are also critical for optimizing overall performance, particularly in the high-end equipment sector characterized by rising demand, including applications in aircraft, engines, and long-range rockets [3]. The design and manufacturing of these components require meticulous selection of materials, well-considered structural design, and integration of advanced forming technologies to achieve optimal performance and reliability. Consequently, the manufacturing technology for complex thin-walled curved surface structures has been identified as a key area for focused development within the broader field of manufacturing technology [4].

With the rapid advancement of aerospace technology, engineers are demanding higher performance from structural designs. Specifically, the need for greater transport loads, improved thrust-to-weight ratios [5], and extended range and component lifespan [6] has accelerated the widespread application of large, complex hyperbolic thin-walled components. These components not only feature intricate geometries but also exhibit advanced technological characteristics across multiple dimensions. The adoption of novel lightweight materials, such as titanium alloys [7] and high-strength aluminum alloys [8], provides exceptional strength-to-weight ratios, enabling systems to minimize weight without compromising structural integrity. Additionally, the introduction of metal matrix and non-metal matrix composites [9] further promotes material lightweighting and functional diversification [10], which are crucial for enhancing the overall efficiency of aerospace vehicles.

The trend toward larger and more integrated structural designs has significantly expanded traditional size ranges, reducing the number of separation surfaces and connection points, and thereby decreasing overall weight. This integrated design philosophy enhances manufacturing efficiency while simultaneously improving the stability and reliability of the structures. As wall thickness continues to decrease, engineers face the challenge of reducing thickness from several millimeters to just one or two millimeters, significantly increasing the technical complexity and process requirements associated with the forming process.

In the aerospace sector, large titanium alloy [11] hyperbolic thin-walled structures are extensively utilized in critical components, including rear fuselage skin panels, fairings, and exhaust systems [12]. TC4 titanium alloy [13] has high specific strength, creep resistance, corrosion resistance, and good weldability. In addition, it has excellent mechanical properties at both room and medium temperatures, as well as good thermal stability up to a maximum service temperature of 400 °C. It is also suitable for use in a wide range of applications. TC4 titanium alloy is widely utilized in mid- to low-temperature components of fourth-generation aircraft and engines in China, Europe, and the United States. Currently, it is one of the most commonly used titanium alloys [14,15] due to its exceptional overall performance in the aerospace sector. The design and manufacturing of these components not only require advanced forming technologies but also the ability to withstand various environmental challenges. The evolving requirements for materials and structures have increased the complexities associated with the forming of thin-walled hyperbolic structures. As a result, the forming technology for large thin-walled hyperbolic structures has emerged as a focal point in current research on plastic-forming technologies [16].

Hyperbolic thin-walled structures primarily take two forms: hyperbolic convex and hyperbolic saddle [17]. These configurations present challenges such as low planar rigidity and a tendency toward instability. Moreover, during flat sheet forming, the material experiences complex tensile and compressive stress states at various locations, which can lead to surface defects such as waviness, wrinkles, and thinning post-formation. The occurrence of these issues not only impacts product durability but also has the potential to affect the aerodynamic performance of critical components, thereby influencing overall flight performance. Therefore, advancing research and technology to enhance the forming processes for thin-walled hyperbolic structures has become a pivotal focus in the field of plastic-forming technologies. For the hot forming of hyperbolic skins, three primary processing methods are commonly employed: small-margin precise positioning coupled hot pressing, pre-thickened stacked forming, and curved edge hot deep drawing [18]. This study focuses on two forming methods: small-margin precise positioning coupled hot pressing and curved edge hot deep drawing. The small-margin coupled hot pressing process involves unfolding the shape of components within a coupled mold using a reference point, and then applying a specific margin at elevated temperatures. This study aims to validate the effectiveness of the hot forming process in optimizing the formability of hyperbolic thin-walled skins. Through the design and implementation of a tailored hot forming methodology, key process parameters are identified and optimized. The final geometries of the hyperbolic saddle and cambered skins are successfully achieved with smooth, defect-free surfaces. Furthermore, the performance degradation of the titanium alloy after thermoforming is limited to within 5% of the original base material, meeting stringent requirements for both profile accuracy and mechanical properties. These results demonstrate the suitability of the process for manufacturing hyperbolic skins in titanium alloy fuselage structural components.

## 2. Materials and Methods

### 2.1. Material and Hyperbolic Structures

**Figure 1:**
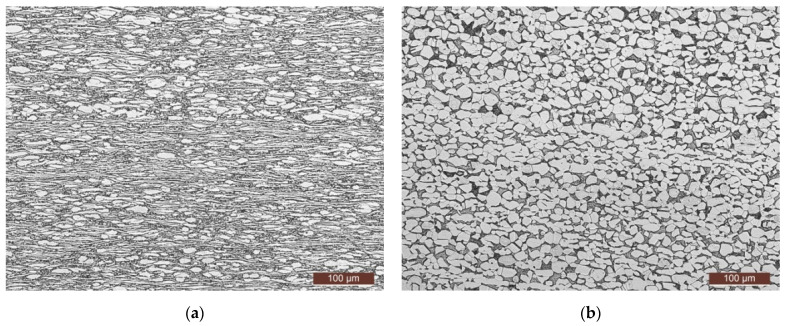
Microstructure of the as-received TC4 titanium alloy along (**a**) longitudinal and (**b**) transverse directions.

The material applied in the present research is a TC4 titanium alloy in the M state [19], with a thickness of 1.2 mm. TC4 titanium alloy is classified as an α + β type titanium alloy, with its microstructure illustrated in Figure 1. TC4 titanium alloy is classified as an α + β type titanium alloy, with its microstructure illustrated in Figure 1, of which the magnification is 200×. This alloy comprises a 6% α-stabilizing element (Al) and a 4% β-stabilizing element (V).

**Figure 2:**
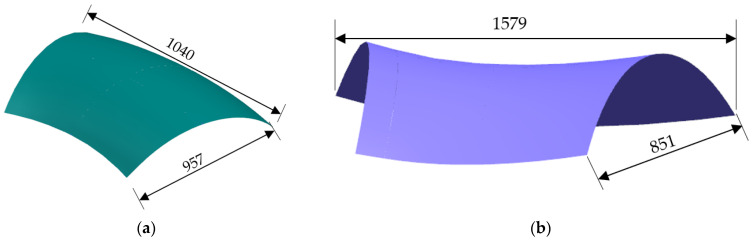
Dimensions of hyperbolic skins: (**a**) convex configuration and (**b**) saddle configuration.

The test component configuration under investigation is a hyperbolic skin structure. The hyperbolic convex configuration is characterized by a consistent curvature in both longitudinal and transverse directions, with varying curvature at different positions and cross-sections. These curves collectively form a positively outwardly convex shape, as illustrated in Figure 2a. In contrast, the hyperbolic saddle configuration exhibits opposing curvature in the transverse direction. Similarly, the curvature at various positions and cross-sections differs, resulting in a surface profile that is bulging laterally and concave longitudinally, resembling a saddle. Consequently, this configuration is referred to as the saddle configuration, as shown in Figure 2b.

**Figure 3:**
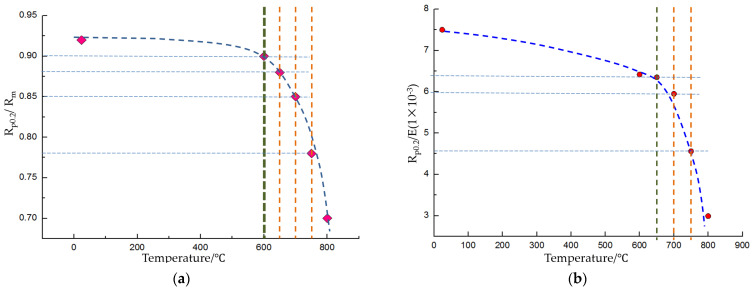
(**a**) Strength-to-weight ratio curve and (**b**) springback curve of TC4 at room and elevated temperatures.

Due to the high elastic modulus, high strength-to-weight ratio, and substantial resistance to deformation of titanium alloys at room temperature, the plastic processing window is limited, making forming particularly challenging, as shown in Figure 3. In contrast, the plasticity of titanium alloys is significantly enhanced at high temperatures above 700 °C, which is the reason that forming processes are commonly employed. The selection of specific parameters is informed by tensile tests of TC4 [20] at both room and elevated temperatures. These tests provide fundamental mechanical performance indicators, including ultimate strength, yield strength, elastic modulus, and elongation, which serve as the basis for the selection of hot forming parameters. The strength-to-weight ratio characterizes the material’s plasticity. In detail, a higher value indicates that the yield strength is closer to the ultimate strength, suggesting that the material is prone to fracture shortly after yielding. Additionally, the ratio of Rp0.2 to E reflects the material’s springback behavior, with a higher value indicating greater springback during forming. The performance characteristics of TC4 at room and elevated temperatures are summarized in Table 1, where R_m_ denotes the tensile strength, R_p0.2_ denotes the yield strength, E denotes the modulus of elasticity, and A denotes the elongation of the material. The data in Table 1 are obtained by conducting uniaxial tensile tests in the present research, of which the procedures are described in Section 2.2. Notably, the strength-to-weight ratio of TC4 at room temperature is 0.94, and the Rp0.2/E ratio is 7.5 × 10^−3^. At 750 °C, the strength-to-weight ratio decreases to 0.75, while the Rp0.2/E ratio drops to 4.5 × 10^−3^, indicating a 20% reduction in strength-to-weight ratio and a 40% reduction in the Rp0.2/E ratio. Considering both cost and forming performance, hot forming at 750 °C is chosen as the optimal process.

### 2.2. Uniaxial Tensile Tests and Hot Forming Processes

Researchers conduct uniaxial tensile tests [21] on both the as-received and annealed materials at temperatures of 700 °C, 750 °C, and 800 °C, utilizing strain rates of 0.002 s^−1^, 0.01 s^−1^, and 0.02 s^−1^. The experiments employ dog-bone-shaped specimens with a gauge length of 20 mm and a width of 6 mm. Once the heating furnace reaches the desired temperature, the specimens are placed in the furnace and soaked for 10 min to ensure uniform temperature distribution throughout each specimen prior to testing. All tests are conducted in an air environment. Following the tensile tests, the specimens are immediately subjected to water quenching for rapid cooling.

**Figure 4:**
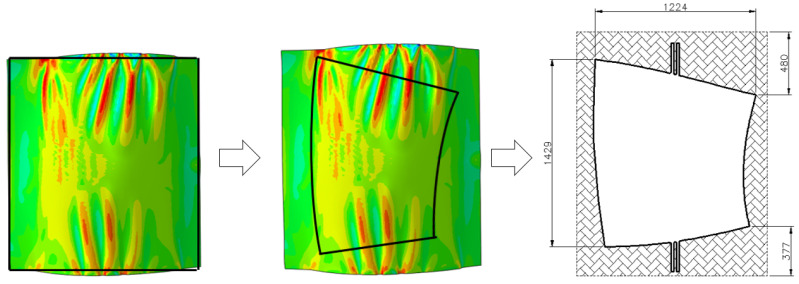
Removal of edge margin with maximum equivalent stress from the sheet material.

**Figure 5:**
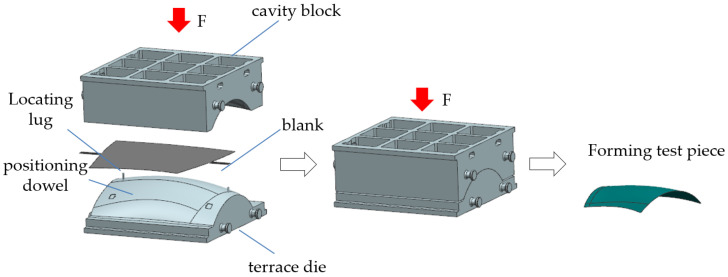
Schematic of the small-margin pin precise positioning coupled hot pressing process.

As shown in Figure 4 and Figure 5, this method utilizes a die with a convex-concave coupling structure to perform pressure coupling forming on the titanium alloy sheet in a heated state. This approach effectively avoids the unnecessary complexity associated with forming intricate profiles, rendering the process relatively straightforward. However, a notable drawback is its inability to mitigate wrinkling and rippling in more complex curved surface formations.

**Figure 6:**
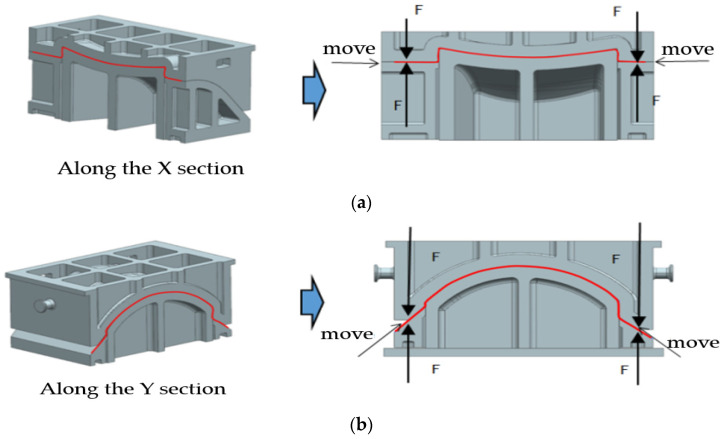
Structure and forming principles of the curved edge hot forming die: (**a**) Along the X section, (**b**) Along the Y section.

**Figure 7:**
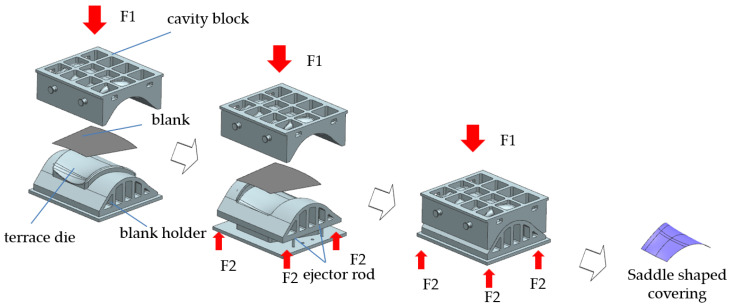
Schematic of the curved edge deep drawing process.

In the hot deep drawing process, a blank holder is utilized to constrain the workpiece, effectively mitigating defects such as instability, wrinkling, and rippling that can occur during forming. This capability enables the deep drawing process to produce components with more complex shapes. However, this technique imposes higher requirements on the equipment, necessitating a design that incorporates an ejection mechanism. The structure of the mold and the principles of the forming process are outlined, as shown in Figure 6 and Figure 7.

In the selection of materials for hot forming dies, silicon-molybdenum ductile iron is used. The hot forming process is conducted on an RX-400T platform heating-type hot forming machine, utilizing K-type thermocouples for temperature measurement. The process parameters for hot forming are detailed in Table 2 and Table 3. After forming, the blank is removed from the die and placed on an asbestos mat for air cooling. Once the surface temperature of the component has cooled to room temperature, a visual inspection is conducted to assess the surface defect status and distribution. Additionally, calipers and other measuring tools are used to evaluate the surface conditions of specimens with varying chord heights and lengths, while a scanner is employed to assess the dimensional accuracy of the specimens.

### 2.3. Microstructure Observation Experiment

The microstructure experiments are conducted using a ZEISS Supra 55 scanning electron microscope to observe the microstructure of TC4 titanium alloy in the T- and L-directions before and after forming. The microscope’s electron beam accelerating voltage is set at 20 kV to provide high resolution, while the system vacuum is 1.43 × 10^−6^ torr, which greatly reduces the scattering of the electron beam and ensures the imaging quality. Meanwhile, the vacuum of the electron gun reaches 1.29 × 10^−9^ torr, which further ensures the stability of electron emission. Under the experimental conditions, the microstructural characteristics of TC4 are obtained.

## 3. Viscoplastic Constitutive Model

To further characterize the hot forming performance of TC4 titanium alloy, a viscoplastic constitutive model is established. The hot deformation process of TC4 titanium alloy involves several physical phenomena, including phase transformation, dynamic recovery, dynamic recrystallization (DRX), spheroidization, and the evolution of dislocations and damaged cavities. The dynamic evolution of the microstructure during deformation has a direct impact on the flow stress behavior. A typical microstructural evolution observed in this context is DRX, which can be simulated using Equations (1) and (2) [22]:

(1) $$\dot{S}=\frac{q_{1}{\left(0.1+S\right)}^{q_{2}}\left(1-S\right){\bar{\rho }}^{2}}{\bar{d}}$$

(2) $${\bar{\rho }}_{c}=a_{1}{{\dot{\varepsilon }}_{p}}^{a_{2}}$$

In the above equations, S represents the recrystallization fraction, $\dot{S}$ denotes recrystallization rate, $\bar{\rho }$ denotes the normalized dislocation density, $\bar{d}$ indicates the normalized grain size, and ${\bar{\rho }}_{c}$ refers to the critical normalized dislocation density. The term ${\dot{\varepsilon }}_{p}$ signifies the plastic strain rate. The other parameters, such as $q_{1}$, $q_{2}$ and $a_{2}$ are material properties that depend on temperature. 

TC4 titanium alloy comprises both α and β phases, with the transformation from α to β occurring at elevated temperatures. This phase transition can be described using the Johnson–Mehl–Avrami–Kolmogorov (JMAK) equation, as illustrated in Equation (3) [23]:

(3) $$f_{\beta }=\exp\left(\gamma \left(T_{trans}-T\right)\right)$$

where $f_{\beta }$ represents the fraction of the β phase, T is the transformation temperature, and γ is a material constant.

The flow behavior of the α and β phases is described by Equations (4) and (5), respectively:

(4) $${\dot{\varepsilon }}_{p,\alpha }={\left(\frac{\sigma /\left(1-D\right)-R-k_{\alpha }}{K_{\alpha }}\right)}^{n_{1}}{\bar{d}}^{-n_{2}}$$

(5) $${\dot{\varepsilon }}_{p,\beta }={\left(\frac{\sigma /\left(1-D\right)-R-k_{\beta }}{K_{\beta }}\right)}^{n_{1}}{\bar{d}}^{-n_{2}}$$

where $n_{1}$, $n_{2}$, $k_{\alpha }$ and $k_{\beta }$ are temperature-dependent material parameters, where $K_{\alpha }$ and $K_{\beta }$ represent the yield stresses of the α and β phases, respectively, where ${\dot{\varepsilon }}_{p,\alpha }$ denotes the plastic strain rate of phase α and ${\dot{\varepsilon }}_{p,\beta }$ denotes the plastic strain rate of phase β. It is assumed that $k_{\beta }$ = 0.8$k_{\alpha }$, $K_{\alpha }$ = 0.9$K_{\beta }$ [24]. According to the rule of mixtures, the total plastic strain rate of the material can be calculated using Equation (6).

(6) $${\dot{\varepsilon }}_{p}={\dot{\varepsilon }}_{p,\alpha }\left(1-f_{\beta }\right)+{\dot{\varepsilon }}_{p,\beta }f_{\beta }$$

where ${\dot{\varepsilon }}_{p}$ denotes the total plastic strain rate.

The flow stress can be calculated using Hooke’s law, as represented in Equation (7).

(7) $$\sigma =\left(1-D\right)E\left(\varepsilon -\varepsilon_{p}\right)$$

where ε denotes total strain and $\varepsilon_{p}$ denotes plastic strain.

The evolution of dislocation density is also influenced by dynamic recrystallization (DRX), which is described in detail as follows.

(8) $$\dot{\bar{\rho }}=A{\bar{d}}^{C_{4}}\left|{\dot{\varepsilon }}_{p}\right|\left(1-\bar{\rho }\right)-C_{1}{\bar{\rho }}^{C_{3}}-C_{2}\bar{\rho }\frac{\dot{S}}{1-S}$$

where A, $C_{1}$, $C_{2}$, $C_{3}$ and $C_{4}$ are temperature-dependent material parameters.

In Equation (8), the first component represents the accumulation of dislocations resulting from strain hardening and dynamic recovery, while the second and final components describe the reduction in dislocation density due to static recovery and dynamic recrystallization (DRX), respectively.

Isotropic hardening is described by Equation (9).

(9) $$R=B{\bar{\rho }}^{0.5}-R_{0}$$

where R represents isotropic hardening, while $R_{0}$ denotes the initial hardening constant, which is equal to $\frac{B}{\sqrt{2}}$, where B is a temperature-dependent material parameter.

During the deformation process, the evolution of damage is described by Equation (10):

(10) $$\dot{D}=d_{1}\left(1-D\right){\left|{\dot{\varepsilon }}_{p,\beta }-{\dot{\varepsilon }}_{p,\alpha }\right|}^{d_{2}}+d_{6}\frac{\cos h\left(d_{3}\varepsilon_{p}\right)}{{\left(1-D\right)}^{d_{4}}}{\dot{\varepsilon }}_{p}^{d_{5}}$$

where D represents damage, while the plastic strain rates for the α and β phases are denoted as ${\dot{\varepsilon }}_{p,\alpha }$ and ${\dot{\varepsilon }}_{p,\beta }$, respectively. The parameters $d_{1}$, $d_{2}$, $d_{3}$, $d_{4}$ and $d_{5}$ are temperature-dependent material parameters.

In Equation (10), the first term represents the growth of voids resulting from the non-cooperative deformation of the α and β phases, while the second term accounts for the nucleation and growth of voids induced by plastic deformation.

Given that DRX affects the average grain size during the deformation process, this study requires the normalization of grain size. This normalization is represented in Equations (11) and (12) [25].

(11) $$\dot{\bar{d}}=u_{1}{\bar{d}}^{-w_{1}}+u_{2}{\dot{\varepsilon }}_{p}{\bar{d}}^{-w_{2}}-u_{3}{\dot{S}}^{w_{3}}{\bar{d}}^{w_{4}}$$

(12) $$\bar{d}=\frac{d}{d_{0}}$$

where d and $d_{0}$ represent the average grain size and the initial average grain size, respectively, while $\bar{d}$ denotes the normalized average grain size. The parameters $u_{1}$, $u_{2}$, $u_{3}$, $w_{1}$, $w_{2}$, $w_{3}$ and $w_{4}$ are temperature-dependent material parameters.

In Equation (11), the first and second components correspond to the growth of static and dynamic grains, respectively, while the final component describes the grain refinement induced by DRX.

## 4. Theoretical Analysis on Thickness Instability

**Figure 8:**
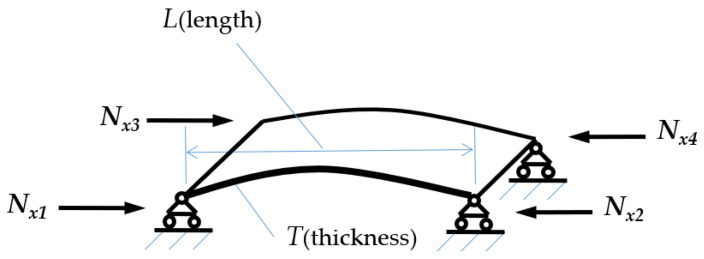
Macroscopic simplified model of compressive instability in sheet material (thin plate four-point simply supported model).

**Figure 9:**
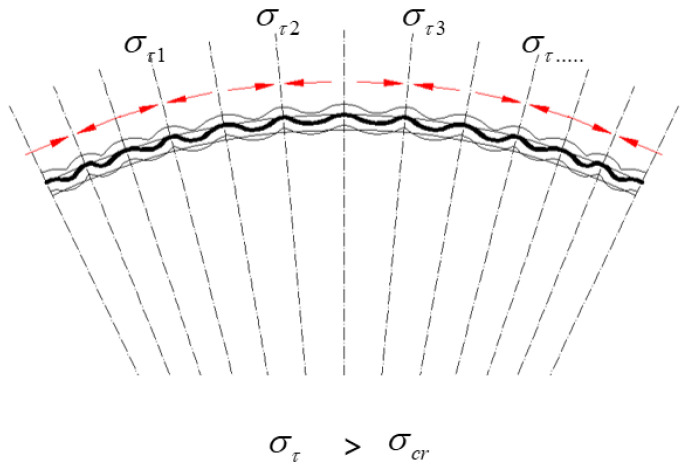
Stress distribution for thickness instability (where $\sigma_{\tau }$ represents tangential compressive stress and $\sigma_{cr}$ denotes critical instability stress).

During the forming process of hyperbolic components, the mold exerts external forces on the sheet material, leading to plastic deformation, with specific regions experiencing compressive stress, as shown in Figure 8. Given the poor stability in the thickness direction of the sheet, when the in-plane compressive stress exceeds the stability limit in that direction, the sheet undergoes wrinkling deformation, a phenomenon known as thickness instability, as shown in Figure 9.

It can be concluded that when the tangential compressive stress in the thin plate exceeds the critical instability stress, the sheet material undergoes bending, resulting in the formation of wrinkles. Consequently, deriving an analytical model for the critical instability stress is essential.

In the field of sheet metal forming, three common challenges—wrinkling, cracking, and springback—are frequently encountered [26]. Among these, springback affects product dimensions and shape accuracy, while cracking has a direct impact on product performance. Wrinkling primarily influences the geometric precision of the product, and its severity can significantly affect overall performance. This study focuses on the typical characteristics of thin-walled, doubly curved skins, which primarily face issues related to forming-induced wrinkling. Building on the theory of compressive instability in sheet materials, an analytical constitutive equation can be derived for the buckling and wrinkling of compressed sheets [27]. Utilizing the energy method, the critical pressure for buckling under compression can be calculated. At this critical state, the bending strain energy of the sheet equals the work completed by the applied pressure. Here, D represents the bending stiffness of the sheet, and the bending strain energy is expressed as follows:

(13) $$\Delta V=\frac{\pi^{4}L_{0}}{8}D\sum_{m=1}^{\infty }\sum_{n=1}^{\infty }a_{mn}^{2}{\left(\frac{m^{2}}{{L_{0}}^{2}}+n^{2}\right)}^{2}$$

(14) $$W=\frac{1}{2}N_{x}\int_{0}^{L_{0}}\int_{0}^{1}{\left(\frac{\partial \omega }{\partial x}\right)}^{2}dxdy=\frac{\pi^{2}}{8L_{0}}N_{x}\sum_{m=1}^{\infty }\sum_{n=1}^{\infty }m^{2}a_{mn}^{2}$$

From Equations (13) and (14), the expression for the critical buckling pressure ${\left(N_{x}\right)}_{cr0}$ can be derived. This critical pressure reaches its minimum value when all coefficients $a_{mm}$ are zero except for $a_{m1}$. The expression for this minimum critical pressure is as follows:

(15) $${\left(N_{x}\right)}_{cr0}=\frac{\pi^{2}{L_{0}}^{2}D}{m^{2}}{\left(\frac{m^{2}}{{L_{0}}^{2}}+1\right)}^{2}$$

This expression indicates that when the sheet metal buckles, there is only one half-wave in the y-direction, while multiple half-waves, denoted as mmm, can occur in the x-direction. The value ${\left(N_{x}\right)}_{cr0}$ is influenced by both the aspect ratio of the sheet and the number of half-waves mmm. Specifically, when $L_{0}$ approaches 1, the minimum critical compressive stress is achieved at m = 1:

(16) $${\left(N_{x}\right)}_{cr0}=\pi^{2}D{\left(\frac{1}{L_{0}}+L_{0}\right)}^{2}$$

As a result, the stiffness variable could be expressed as follows:

(17) $$D_{N}=\frac{N_{x}}{{\left(N_{x}\right)}_{cr0}}D$$

According to the theory of thin plate bending shown in Equations (18)–(22). The expression for the equivalent stress could be derived by Equation (23), where z denotes the coordinate in the thickness direction of the sheet material within the local coordinate system.

(18) $$M_{x}=-D_{N}\left(\frac{\partial^{2}\omega }{\partial x^{2}}+\mu \frac{\partial^{2}\omega }{\partial y^{2}}\right),M_{y}=-D_{N}\left(\frac{\partial^{2}\omega }{\partial y^{2}}+\mu \frac{\partial^{2}\omega }{\partial x^{2}}\right)$$

(19) $$M_{xy}=M_{yx}=-D_{N}\left(1-\mu \frac{\partial^{2}\omega }{\partial x\partial y}\right)$$

(20) $$Q_{x}=-D_{N}{\left(\frac{\partial y}{\partial x}\right)}^{2}\omega ,\;Q_{y}=-D_{N}{\left(\frac{\partial x}{\partial y}\right)}^{2}\omega$$

(21) $$\sigma_{x}=\frac{12M_{x}}{t^{3}}z,\;\sigma_{y}=\frac{12M_{y}}{t^{3}}z,\;\sigma_{z}=-2q{\left(\frac{1}{2}-\frac{z}{t}\right)}^{2}\left(1+\frac{z}{t}\right)$$

(22) $$\tau_{xy}=\tau_{yx}=\frac{12M_{xy}}{t^{3}}z,\;\tau_{xz}=\frac{6Q_{x}}{t^{3}}\left(\frac{t^{2}}{4}-z^{2}\right),\;\tau_{yz}=\frac{6Q_{y}}{t^{3}}\left(\frac{t^{2}}{4}-z^{2}\right)$$

(23) $$\bar{\sigma }=\sqrt{\frac{1}{2}\left[{\left(\sigma_{x}-\sigma_{y}\right)}^{2}+{\left(\sigma_{y}-\sigma_{z}\right)}^{2}+{\left(\sigma_{z}-\sigma_{x}\right)}^{2}+6{\left({\tau^{2}}_{xy}+{\tau^{2}}_{yz}+{\tau^{2}}_{zx}\right)}^{2}\right]}$$

In sheet forming processes, the primary stresses are denoted as $\sigma_{x}$, $\sigma_{y}$ and $\tau_{xy}$, while the secondary stresses $\sigma_{z}$, $\tau_{xz}$ and $\tau_{yz}$ can be disregarded for calculation purposes. By simultaneously considering the equations above and varying the parameters x, y, and z, it is possible to determine the maximum equivalent stress ${\bar{\sigma }}_{\max}$, which depends solely on the unknown variable $N_{x}$. The critical instability pressure at which wrinkling of the sheet material occurs is expressed as follows:

(24) $$N_{cr}=\frac{\pi^{2}ED}{L^{2}}$$

where E represents the elastic modulus, L denotes the length of the sheet.

D is the moment of inertia and its value:

(25) $$D=\frac{Et^{3}}{12(1-\mu^{2})}$$

The final expression for the critical buckling stress of the sheet under compression is derived as follows:

(26) $$\sigma_{cr}=\frac{E\pi^{2}t^{2}}{12(1-\mu^{2})L^{2}}$$

In this context, $\sigma_{cr}$ represents the critical compressive stress of the sheet; t denotes the sheet thickness; μ is the Poisson’s ratio; L is the sheet length; and E is the generalized reduction coefficient. From this expression, it is evident that the critical buckling stress of the sheet is directly proportional to the square of the reduction coefficient E and the square of the sheet thickness t while being inversely proportional to the square of the sheet length L. Furthermore, it is crucial to recognize that the instability of the sheet under compression is predicated on the absence of loading in the Z-direction. If sufficient stress $\sigma_{z}$ is applied in this direction, the sheet will remain stable. Based on this analysis, three critical parameters determining the thick-direction instability of the skin are identified: boundary dimensions, material thickness, and the presence of constraints in the thickness direction.

## 5. Results and Discussions

### 5.1. Uniaxial Tensile Test Results

**Figure 10:**
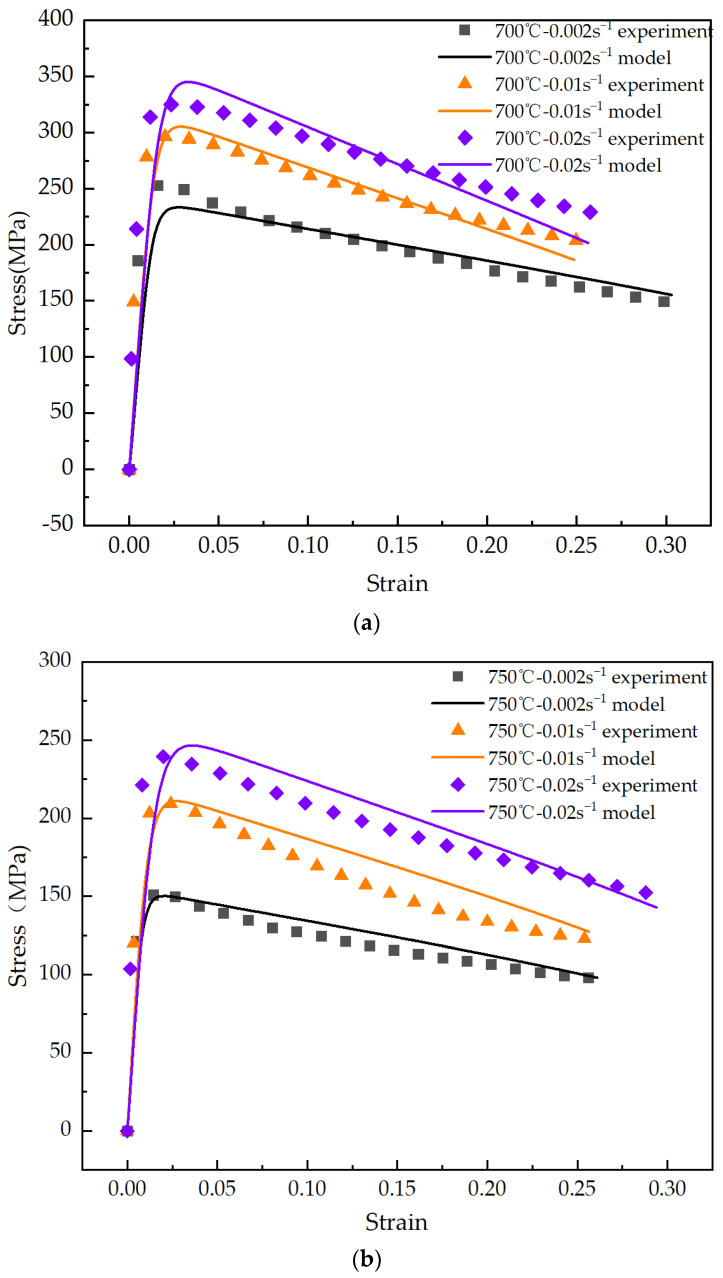

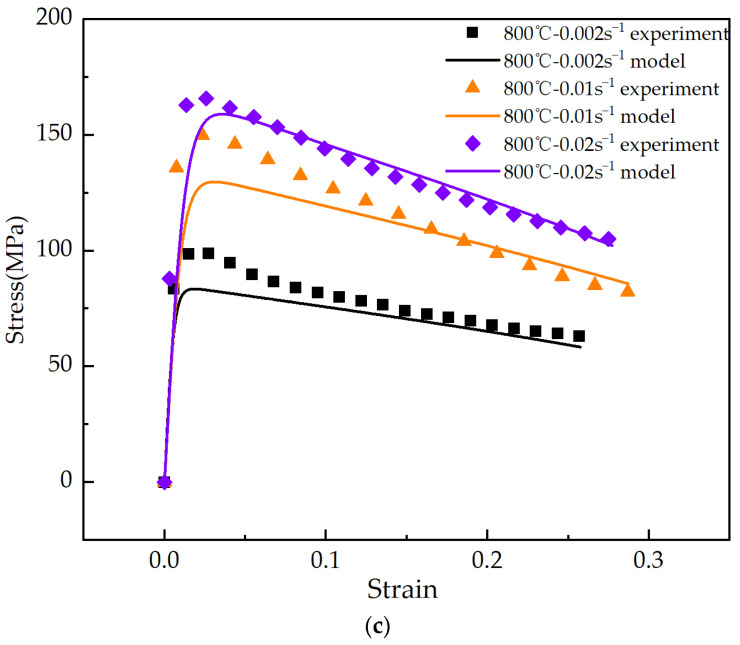
Experimental and predicted stress-strain curves of TC4 titanium alloy at (**a**) 700 °C, (**b**) 75 °C, and (**c**) 800 °C.

Tensile tests were performed on TC4 titanium alloy at temperatures of 700 °C, 750 °C, and 800 °C. For each temperature, three distinct strain rates of 0.002, 0.01, and 0.02 s^−1^ were employed to assess the variation in the stress-strain curves. The established constitutive model was utilized for curve fitting, with the results presented in Figure 10.

It is shown that the predictions generated by the established viscoplastic constitutive model exhibit a high degree of correlation with the experimental data, indicating effective fitting of the stress-strain curves for TC4 titanium alloy. This model successfully captures the deformation behavior of TC4 titanium alloy during tensile testing. At 700 °C, the alloy experiences significant stress, characterized by a rapid decrease in true stress as true strain increases. Conversely, at 750 °C and 800 °C, the stress-strain curves reveal a more gradual decline in true stress with increasing true strain, signifying enhanced plasticity. Notably, the sensitivity to strain rate at 800 °C is higher than that observed at 700 °C and 750 °C, leading to a substantial influence on stress at equivalent strain levels. Considering the experimental costs, 750 °C is ultimately selected as the optimal temperature for conducting thermal forming experiments on the titanium alloy double-curved skin.

### 5.2. Numerical Simulation Results of Hot Forming

#### 5.2.1. Numerical Simulation Results of Convex Double-Curved Skin

This section presents a numerical simulation result of the hot forming process for a convex double-curved skin, conducted using ABAQUS version 2022 software. The simulation is based on a rectangular titanium alloy blank undergoing coupled hot pressing. The finite element modeling includes two structures: the hyperbolic convex skin and the hyperbolic saddle skin, both modeled as three-dimensional deformable components. Quadrilateral elements are used to mesh the hyperbolic convex skin and hyperbolic saddle skin, which comprises 43,967 and 63,524 elements, respectively. The material properties of TC4 shown in Table 1 are input to the Finite Element model. In addition, the friction coefficient of TC4 is defined as 0.42 [28], and the heat transfer coefficient is defined as a function of pressure ranging from 0.8 to 5 kW/m^2^K [29]. The analysis aims to assess the sheet forming conditions and to predict potential defect states prior to the actual forming process.

**Figure 11:**
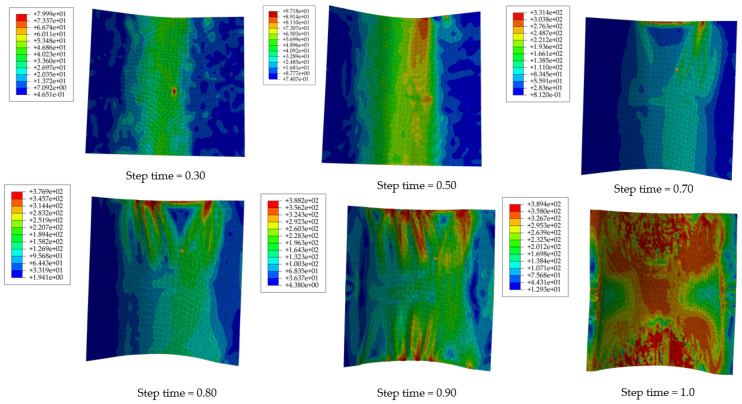
Numerical simulation results of hot forming for convex double-curved skin.

The finite element analysis results of the hyperbolic cambered skin are shown in Figure 11.

**Figure 12:**
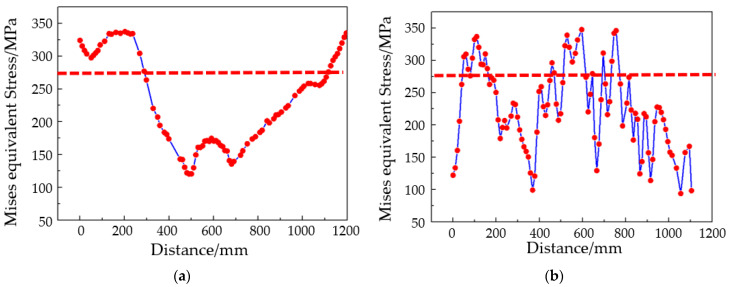
Convex double-curved skin equivalent stress curves along (**a**) the x direction (long edge) and (**b**) the y direction (short edge).

Figure 12 shows the equivalent stress analysis along the x and y directions. From the equivalent stress data during the sheet forming process, it can be observed that at a step time of 0.4, the maximum equivalent stress occurs at the center position. At a step time of 0.8, the maximum equivalent stress shifts to the edge, resulting in a decrease in equivalent stress at the center. At a step time of 0.9, the peak equivalent stress at the edge reaches 388 MPa, indicating significant wrinkling of the material. Finally, at a step time of 1.0, after the male and female dies make contact, the maximum equivalent stress increases to 389 MPa, with severe wrinkling occurring at the short edge of the skin and ripple formation along the long edge.

#### 5.2.2. Numerical Simulation Results of Hyperbolic Saddle Skin

**Figure 13:**
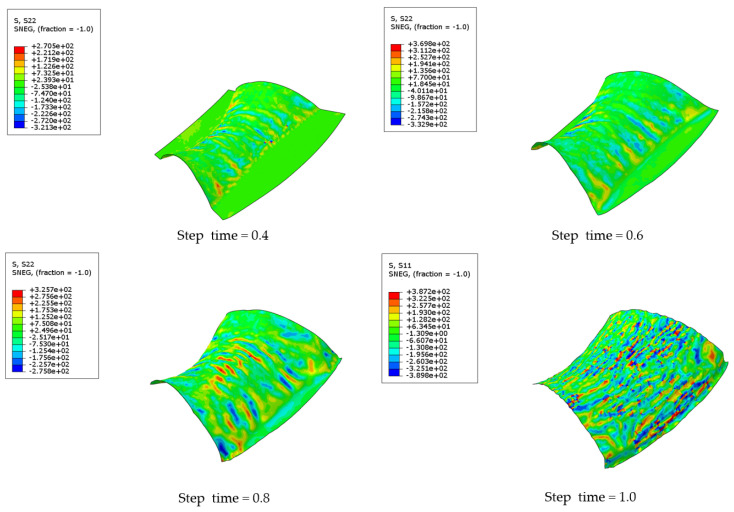
Numerical simulation results of hot forming for hyperbolic saddle skin.

Analysis of the equivalent stress during the sheet forming process reveals several key observations. At a step time of 0.4, the concave surface begins to form, with significant compressive stress concentrated in the center region, yielding a maximum equivalent stress of 300 MPa. At this stage, the sheet exhibits waviness in a direction perpendicular to the saddle. As step time progresses to 0.6, the waviness continues to intensify, resulting in a maximum equivalent stress of 319 MPa. At a step time of 0.8, the maximum equivalent stress shifts to the edge of the saddle bulge, where tensile stress predominates, leading to a gradual reduction in waviness. Finally, at a step time of 1.0, the maximum equivalent stress disperses across multiple regions of the skin, with the sheet displaying both longitudinal waviness and a tendency toward wrinkling, particularly along the edges of the saddle’s convex surface. The finite element analysis results of the hyperbolic saddle skin are shown in Figure 13.

**Figure 14:**
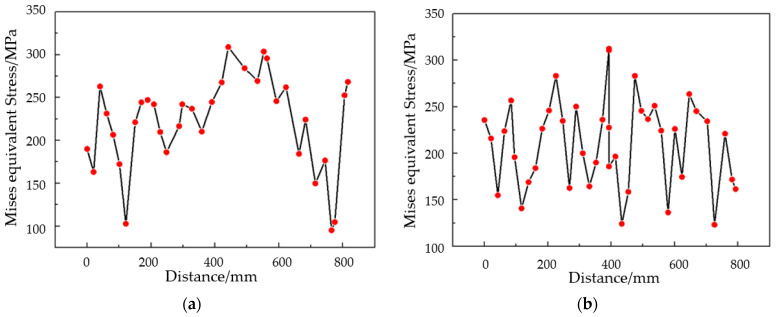
Equivalent stress curves along (**a**) the x direction (long edge) and (**b**) the y direction (short edge).

Figure 14 presents the equivalent stress distribution at the center position of the sheet at a step time of 1.0. The simulation analysis indicates that the wrinkling of the formed sheet is closely associated with its equivalent stress at this state and the critical instability stress. During the hot forming process of the convex double-curved skin, the equivalent stress at the edges of the titanium alloy exceeds the critical instability stress, resulting in compressive instability and subsequently leading to waviness and wrinkling. In a similar manner, for the saddle-shaped skin, the equivalent stress in the central region surpasses the critical instability stress, which is the primary cause of wrinkling and waviness. However, it is noteworthy that, unlike the convex skin, the peak equivalent stresses in the two directions of the saddle-shaped skin exhibit an irregular and dispersed distribution, whereas the stresses in the convex skin are primarily concentrated at the two short edges. Based on these findings, it can be concluded that reducing the equivalent stress at the edges of the convex double-curved skin and enhancing the resistance to instability stress in the saddle-shaped skin are effective strategies for mitigating wrinkling issues.

### 5.3. Defect States and Distribution Patterns

**Figure 15:**
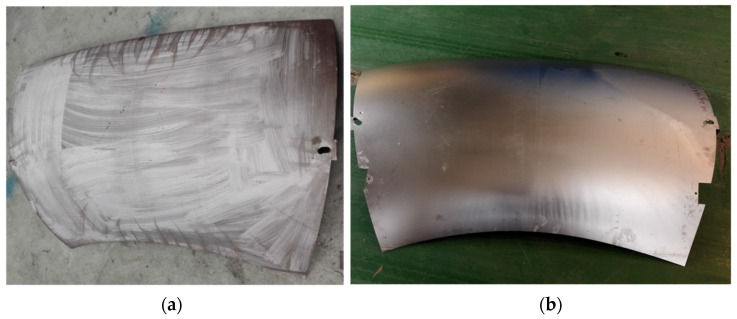
The formed convex double-curved skin: (**a**) small allowance coupled with hot pressing and (**b**) hot deep drawing.

**Figure 16:**
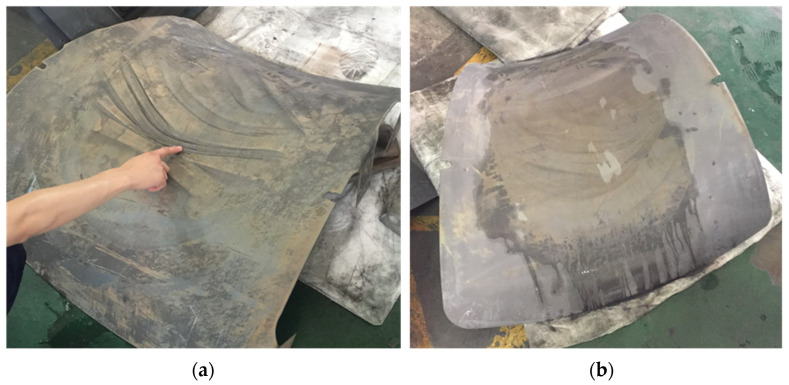
The formed saddle-shaped double-curved skin: (**a**) small allowance coupled with hot pressing and (**b**) hot deep drawing.

Figure 15 and Figure 16 present the surface quality of the convex double-curved and saddle-shaped skin components fabricated using a small allowance pin-locating coupled hot pressing and hot deep drawing process. The results indicate that the convex double-curved skin exhibits waviness along its outer edges. In contrast, the saddle-shaped skin displays pronounced waviness in the central region, with larger undulations. Some of these waves have folded into small rounded corners under the pressure exerted by the male and female dies, significantly affecting the dimensional accuracy of the specimens. This waviness may also adversely impact the performance characteristics, which will be evaluated in subsequent performance tests. Notably, the surface quality of the hot deep-drawn convex double-curved skin has shown considerable improvement, with only localized waviness evident outside the specified allowance, while the rest of the surface remains smooth and of high quality.

**Figure 17:**
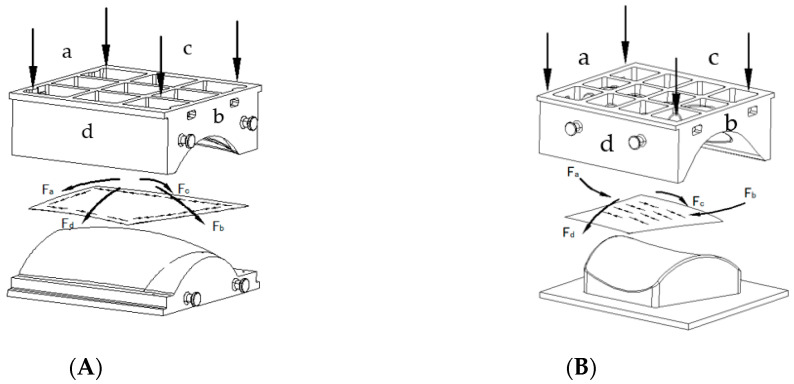
The stress state of the plates during the forming of the doubly curved convex skin and saddle skin using the coupled hot pressing process: (**A**) convex skin and (**B**) saddle skin.

In the case of the double-curved convex skin, during the downward pressing on the platform, the central area of the blank first contacts the convex die, while the edges contact the concave die. As the concave die descends, it exerts pressure F on the blank, causing the material at the edges to experience tensile stresses. Under the influence of the convex surface of the die, the blank bends downward in these four directions, inducing compressive stresses that force the material at the edges to contract. Consequently, the edges of the material are subjected to inward-directed pressure along each edge. As the gap between the convex and concave dies decreases, the compressive stress at the edges increases until it exceeds the yield stress of the material in the thickness direction under the given conditions. This results in the formation of wrinkles and buckles at the edges, as illustrated in Figure 17A. When the convex and concave dies are coupled, initial mild wrinkling can be flattened; however, more significant wrinkles may evolve into further compression of the material, potentially leading to material stacking.

For the saddle-shaped skin, the apex at the ends a and b of the blank contacts the convex die first, as shown in Figure 17. As the concave die continues its descent, the apexes at ends a and b bend outward while the blank experiences tension along the c and d directions and compression along a and b. As the convex and concave dies approach each other, the edge surfaces at a and b first contact the convex die, followed by the c and d edge surfaces. However, the central region remains in a state of tensile and compressive stress. As these tensile-compressive stresses intensify, yielding occurs along a and b, ultimately resulting in the formation of waves and wrinkles.

When forming the doubly curved convex skin using the hot deep drawing process, the plate is initially constrained against the four sides of the die by the clamping ring. This constraint prevents the free movement of the plate, compelling it to flow inward circumferentially under the applied clamping force. If the clamping force is inadequate and fails to exceed the yield strength of the plate, wrinkles will still form around the edges. Conversely, if the clamping force is excessively high, the plate will be unable to flow, resulting in localized stretching, material thinning, or even cracking. Experimental results indicate the presence of some local waviness on the convex skin, suggesting that the clamping force was slightly insufficient; thus, future experiments should aim to increase the clamping force.

**Figure 18:**
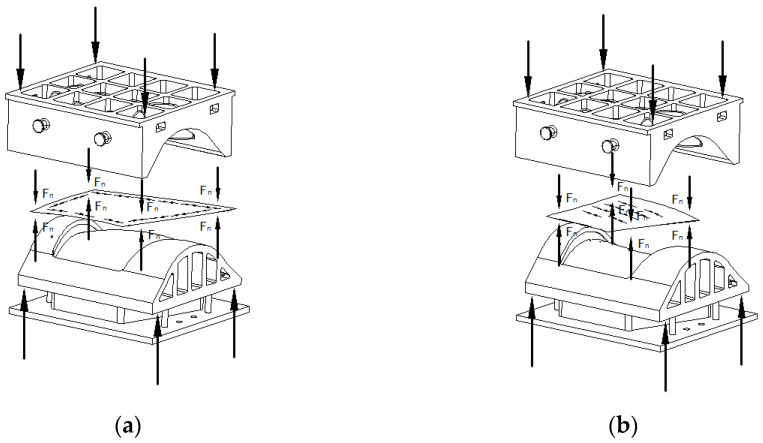
The clamping diagram of the plates during the forming of the doubly curved convex skin and saddle skin using the deep drawing process: (**a**) convex skin and (**b**) saddle skin.

For the doubly curved saddle skin, the same clamping ring and die arrangement is employed to secure the plate, as shown in Figure 18. During the forming process, the plate experiences tensile stress circumferentially, and the edges exhibit a tendency to wrinkle due to compressive stresses. However, the clamping force effectively suppresses this wrinkling, resulting in a smooth surface finish on the part. In summary, the application of clamping force mitigates the instability of the plate during the forming process, allowing it to maintain a smooth profile until the coupling occurs. Both the doubly curved saddle and convex skins formed using these two processes display similar surface defect distributions and conditions, differing primarily in severity. This observation indicates that the clamping structure in the deep drawing process does not alter the force state of the blank. Instead, it applies additional constraining forces to suppress instability, thereby enhancing the surface accuracy of the formed parts.

In comparing the hot forming results, the surface deep drawing process demonstrates superior forming accuracy relative to the coupled hot pressing process. This enhancement can be attributed to the comprehensive constraints established around the blank during deep drawing, which effectively limits the instability and warping of the material. Furthermore, the edges of the blank formed during the deep drawing process generate local flanges, which inadvertently mitigate rebound deformation during the cooling phase after removal from the die.

Conversely, in the small allowance coupled hot pressing process, although the warping of the blank edges is ultimately compressed to conform to the die shape, some rebound still occurs upon removal, which adversely affects the accuracy of the test plate. Analysis of the physical images of the formed components reveals that both the small allowance coupled hot pressing and the surface flanging processes exhibit the characteristic of having higher central areas with relatively lower edge precision. This observation arises from the fact that, in both configurations, the edges are in a relatively free state after forming. As a result, the free edges experience some deformation during the cooling process, while the central region, constrained by the surrounding material, is less susceptible to deformation, thereby maintaining higher accuracy.

### 5.4. Comparison of Surface Accuracy After Forming

**Figure 19:**
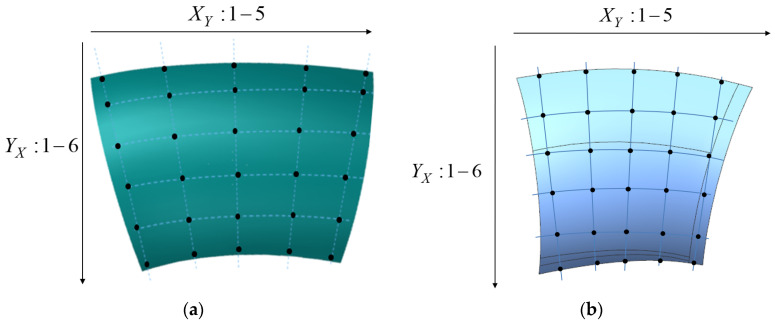
Selection of the points of the hyperbolic skin: (**a**) convex skin; (**b**) saddle skin.

**Figure 20:**
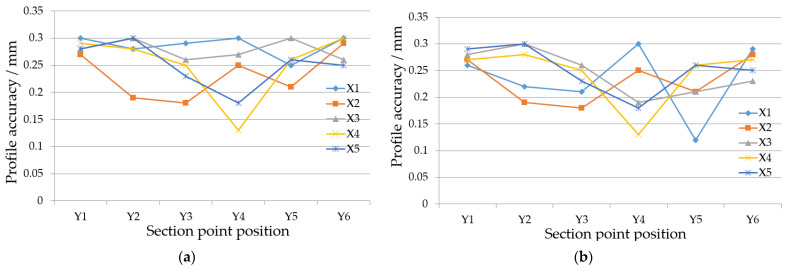
Accuracy of each point’s corresponding profile: (**a**) convex skin; (**b**) saddle skin.

The surface accuracy of the two configurations of skins formed using the surface deep drawing process was assessed by measuring points in both the x and y directions, as illustrated in Figure 19 and Figure 20. As a result of theoretical analysis through the viscoplastic constitutive model and skin stability model as well as the application of optimal temperature of 750 °C, the precision across all measured points on both configurations ranges from 0.1 mm to 0.3 mm, indicating a high level of forming accuracy. For the hyperbolic convex configuration, the results indicate that surface accuracy is lower at the edges compared to the central region, which exhibits higher precision. The edges, being in an unconstrained state, experience significant localized tensile and compressive stress, leading to some rebound after forming. Similar to convex skins, saddle skins also exhibit a high overall profile accuracy. The larger edge allowance and the presence of a pronounced flange enhance the stiffness of the surface, effectively suppressing rebound deformation.

### 5.5. Chord Height, Chord Length and Wrinkling

A detailed analysis of Figure 11 illustrates the variations in equivalent stress during the coupled hot-press forming of the hyperbolic convex skin. In Figure 11, at a step time of 0.3, the maximum equivalent stress occurs in the central region, measuring 42 MPa, while the stress at the two side edges is nearly zero due to lack of deformation. At a step time of 0.5, it shows that the maximum equivalent stress increases to 80 MPa, with the undeformed regions on both sides still exhibiting an equivalent stress of 0. As deformation progresses, the equivalent stress in the upper right area continues to rise. At a step time of 0.7, the maximum equivalent stress reaches 331 MPa, resulting in the onset of wrinkling in this area. With further deformation to a step time of 0.8, the maximum equivalent stress increases to 377 MPa. At a step time of 0.9, severe wrinkling appears on both sides, with the maximum equivalent stress reaching 388 MPa. At a step time of 1.0, although the molds have fully engaged, slightly reducing the wrinkling, it does not completely eliminate it, and new wrinkles emerge on the other sides, with the maximum equivalent stress reaching 389 MPa.

From a step time of 0.7, the equivalent stress in the sheet exceeds the yield strength, leading to the formation of ripples and wrinkles. The measured chord lengths are $L_{a-b}$ = 1048.1 mm and $L_{c-d}$ = 788.5 mm, with chord heights $H_{a-b}$ = 21.4 mm and $H_{c-d}$ = 164 mm. During further coupling at a step time of 0.8, the chord lengths are $L_{a-b}$ = 1060.5 mm and $L_{c-d}$ = 798.3 mm, with chord heights $H_{a-b}$ = 25.5 mm and $H_{c-d}$ = 168 mm. Ignoring the influence of torsional surface effects, we calculate the ratio of total chord height to total chord length for the hyperbolic convex configuration as follows:

$$T_{0.7}=(H_{a-b}+H_{c-d})/(L_{a-b}+L_{c-d})=0.101,\;T_{0.8}=(H_{a-b}+H_{c-d})/(L_{a-b}+L_{c-d})=0.104$$

As the coupling process continues, when T = 0.113, significant wrinkling occurs at the edges of the blank, leading to local compression and stacking.

Through experiments conducted on the hot forming process, we derived parameters related to the thickness instability of the hyperbolic skin. The study reveals that the ratio of the total chord height to the total chord length serves as a critical parameter for determining whether wrinkling will occur in the skin during forming. This ratio is designated as T. When T ≤ 0.08, there is no risk of wrinkling; conversely, when T > 0.08, the skin is likely to experience thickness instability, necessitating measures to mitigate this instability.

Subsequently, process experiments were conducted to validate the relationship between the sum of chord heights and the sum of chord lengths of actual hyperbolic skins and their propensity to wrinkle. Three outer convex skins and saddle skins are selected, each with a uniform thickness of 1.2 mm. After the coupling hot pressing process, the chord lengths and chord heights are measured to calculate their ratio, T. Concurrently, the occurrence of wrinkles is observed, resulting in the following Table 4:

Therefore, it can be concluded that for double-curved convex and saddle skins with a thickness of 1.2 mm, maintaining a ratio of the sum of chord heights to the sum of chord lengths within 0.08 ensures that the application of a small allowance precision positioning coupled with hot pressing process yields formed specimens with good surface quality, free from wrinkling. Conversely, if this ratio exceeds 0.08, the components are likely to exhibit wrinkling and rippling.

### 5.6. Microstructure and Properties Before and After Forming

The convex skin exhibiting the best performance from the hot deep drawing process is selected for analysis. From the surplus material area, tensile test and metallographic samples are extracted for tensile performance evaluation and microstructural examination. These results are then compared with the mechanical properties and microstructure of the original sheet material. Samples 1#, 2#, and 3# correspond to the metallographic structures at angles of 45°, transverse (T), and longitudinal (L), respectively. The mechanical properties and microstructures are summarized in Table 5.

**Figure 21:**
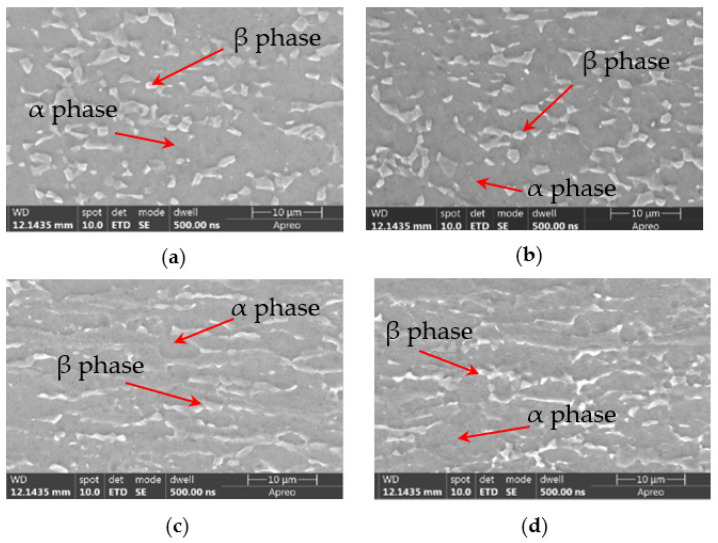
Microstructure of TC4 titanium alloy: (**a**) along transverse direction before forming, (**b**) transverse direction after forming, (**c**) longitudinal direction before forming, and (**d**) longitudinal direction after forming.

The microstructures of the original material in the transverse (T) and longitudinal (L) directions were compared with those of the formed material in the respective directions. The results are shown in Figure 21 with a magnification of 1000×.

The mechanical properties of the formed material are comparable to those of the original base material. Metallographic analysis indicates that the microstructure post-forming retains an equiaxed α + β phase structure, with no significant alterations observed. The material undergoes a degree of plastic deformation, which introduces a high-temperature deformation softening effect. This phenomenon results in the transformation of irregular intergranular β phases into spherical β phases. Consequently, there is a slight enhancement in the material’s plasticity, evidenced by an increase in elongation.

By calculating and analyzing the changes in mechanical properties before and after forming in this experiment, it is found that the Rm of TC4 decreased by 2% and Rp decreased by 1%. In another study of hot stamping of TC4 [30], the decrease in Rm and Rp before and after hot forming of titanium alloys is 6% and 7%, respectively. Additionally, the post-form microstructures of TC4 of these two studies are similar, containing equiaxed α + β phase structure.

By comparing the mechanical properties and microstructure before and after forming, it is verified that a hot deep drawing of TC4 at the temperature of 750 °C is optimal. In future work, a comprehensive study will be conducted to compare and analyze the microstructure and mechanical properties of TC4 under different forming conditions.

## 6. Conclusions

This study investigates the relationship between the instability characteristics and defect features of thin-walled double-curved skins, constructs a constitutive model for TC4 titanium alloy, and conducts uniaxial tensile tests. Additionally, a skin instability model is developed to elucidate the three critical parameters influencing the thickness-wise instability of the skin. For both the convex double-curved skin and the saddle double-curved skin, small-margin coupling hot pressing and surface edge forming processes are employed, respectively. The results explore the manifestation and distribution patterns of forming defects for different shapes and compare profile accuracy post-formation. The relationship between the sum of chord heights and the sum of chord lengths, as well as the occurrence of waviness, is analyzed, along with observations of the metallographic structure of the titanium alloy skin before and after forming. The following conclusions are drawn:

(1) The viscoplastic constitutive model accurately fits the stress-strain curves of the titanium alloy, demonstrating a high degree of correlation with experimental results. The model effectively predicts the performance variations of TC4 titanium alloy during hot forming, indicating that conducting hot forming tests at 750 °C is appropriate.
(2) The convex double-curved configuration is prone to wrinkling at the edges, with the wrinkling direction perpendicular to the edge direction. In contrast, the saddle configuration tends to develop wrinkles in the center, with the wrinkling direction nearly perpendicular to the saddle’s front and rear. The underlying cause of wrinkling in both cases is that the local compressive stress in the sheet exceeds the critical instability stress. Wrinkling can be mitigated by reducing edge forming stress and increasing resistance to instability loads. The identified stress instability model reveals the mechanism of thickness-wise instability: the critical instability stress is directly proportional to the square of the sheet thickness and inversely proportional to the square of the boundary dimensions.
(3) The skin instability model indicates that thickness-wise instability is macroscopically related to the tangent length of the skin, material thickness, and boundary conditions. It establishes that the ratio of the sum of chord heights to the sum of chord lengths is a key parameter determining whether wrinkling occurs. When the ratio T ≤ 0.08, the sheet will not experience thickness-wise instability; when T > 0.08, the sheet will wrinkle, necessitating instability suppression measures.
(4) The profile accuracy of the convex double-curved skin shows significant fluctuations, with the accuracy in the middle position being superior to that at the edges. In contrast, the saddle double-curved skin exhibits smaller fluctuations in profile accuracy, with no apparent regularity. This discrepancy arises because the material in the middle position is constrained circumferentially, making it less susceptible to deformation, while the edge position is free, leading to a higher likelihood of rebound deformation.
(5) After forming TC4 titanium alloy thin plates at 750 °C, no significant changes in the microstructure are observed. Due to the high-temperature softening effect, a slight improvement in material plasticity is noted post-formation, evidenced by a marginal increase in elongation.

## Figures and Tables

**Table 1:** Uniaxial tensile properties of TC4 at room and elevated temperatures.

Temp./°C	Strain Rate/s^−1^	R_m_/MPa	R_p0.2_/MPa	A/%	E/GPa
24	0.1	923	843	17.20	112.5
600	0.1	489.67	414.39	49.56	64.5
650	0.1	399.90	361.06	63.36	49.0
700	0.1	320.65	273.59	79.52	43.8
750	0.1	227.12	169.81	135.24	42.7
800	0.1	140.81	99.22	239.88	33.2

**Table 2:** Coupled hot pressing process parameters.

Type of Skin	Forming Temp./°C	Forming Load/t	Holing Time/min	Blank Margin/mm
Convex skin	750	80	30	50
Saddle skin	750	80	30	50

**Table 3:** Deep drawing process parameters.

Type of Skin	Forming Temp./°C	Forming Load/t	Holing Time/min	Blank Margin/mm
Convex skin	750	100	20	30
Saddle skin	750	100	30	30

**Table 4:** Relationship between chord length, chord height, and wrinkling of skins.

Skin No.	Longitudinal Chord Length /mm	Longitudinal Chord Height /mm	Transverse Chord Length /mm	Transverse Chord Height /mm	T Value	Wrinkling
Convex skin1#	1061.5	24.5	788.8	166.8	0.103	Affirmative
Convex skin2#	1172.0	68.4	941.2	162.0	0.109	Affirmative
Convex skin3#	1253.0	38.5	673.3	113.6	0.079	Negative
Saddle skin1#	675.8	26.4	794.5	171.7	0.135	Affirmative
Saddle skin2#	571.7	36.2	832.2	163.7	0.143	Affirmative
Saddle skin3#	1108.6	30.7	660.2	111.5	0.080	Negative

**Table 5:** Mechanical properties of TC4 titanium alloy before and after forming.

No.	R_m_/MPa	R_p0.2_/MPa	A/%	E/GPa
As-received-1#	1025	967	18.1	110
As-received-2#	1011	943	17.3	109
As-received-3#	1017	955	17.8	110
750 °C-1#	1009	965	22.5	104
750 °C-2#	986	937	22.3	105
750 °C-3#	996	940	23.1	107

## Data Availability

The original contributions presented in this study are included in the article. Further inquiries can be directed to the corresponding author.
